# Novel Approach to Improving Knee Range of Motion in Arthrogryposis with a New Working Classification

**DOI:** 10.3390/children8070546

**Published:** 2021-06-24

**Authors:** David S. Feldman, Troy J. Rand, Aaron J. Huser

**Affiliations:** Paley Advanced Limb Lengthening Institute, St. Mary’s Hospital, West Palm Beach, FL 33407, USA; trand@paleyinstitute.org (T.J.R.); ahuser@paleyinstitute.org (A.J.H.)

**Keywords:** arthrogryposis, knee flexion contracture, arc of motion

## Abstract

Arthrogryposis multiplex congenita (AMC) is a rare condition defined as contrac-tures in multiple joints. Surgical interventions for severe knee flexion contractures have included posterior release, distraction and extension with external fixation and distal femoral extension osteotomies. These operations have been able to achieve knee extension, but not increase the range of motion. The purpose of this study was to review our experience with peroneal nerve decompression, posterior knee release and proximal femoral shortening. We retrospectively reviewed the medical charts and radiographs of all patients with a diagnosis of arthrogryposis who underwent aforementioned procedure. There were 39 patients with 73 knees included in the analysis with a mean follow-up of 21 months. The mean preoperative arc of motion was 45° and last followup arc of motion was 79° (*p* < 0.0001). The mean last followup flexion contracture was 8° (*p* < 0.0001). Additional subanalyses were performed on those with followup greater than 24 months and those with flexion contractures >60°; there were no differences found in these groups. This study demonstrates that it is possible to achieve a functional range of motion of the knees in patients with AMC while improving ambulatory function.

## 1. Background

Arthrogryposis multiplex congenita (AMC) is a rare condition defined as contrac-tures in multiple joints affecting 1:3000–5000 live births [1,2] Over 400 different conditions have been described that include multiple joints with contracture and that fall under the umbrella of AMC [1]. Hip and knee flexion contractures in arthrogrypotic patients can be the limiting factors in a patient’s ability to ambulate [3].

Treatment of these contractures have included: non-operative therapy, osteotomies of the hips/knees, posterior releases of the knee and gradual soft-tissue lengthening with external fixation [4,5,6,7,8,9,10,11,12]. Historically it has been demonstrated that treatment can redirect the arc of motion in AMC; however, it has not been shown that treatment can actually increase the total arc of motion [4,5,6,7,8,9,10,11,12]. If a patient has a knee flexion contracture of 90° with an arc of motion of 15°, then redirection of this arc so that the patient can stand may actually take away their ability to sit. The goal of our treatment is to increase the range of motion to be more inclusive of activities rather than to trade standing for sitting.

The purpose of this study is to describe a single stage novel treatment for the ar-throgrypotic flexion deformity of the knee and hip that increases the arc of motion of the knee. We present the results regarding the procedure, complications, ambulation achieved, and outcomes at 6 months and at 24 months. As well, we describe a new classification to guide treatment.

## 2. Methods

Institutional Review Board approval was obtained from Metrowest Medical Center (IRB# 2021–029) on 24th March 2021. A retrospective chart and radiographic review was performed on all patients seen in our institution with a diagnosis of AMC from October 2015 through September 2020. Patients were identified and classified into three types based on the status of their knee. Type 1 is defined as a flexion contracture (lacking >30° from full extension, with an arc of motion >45°) with a modifier for quadriceps function: present (A) or absent (B). Type 2 is defined as an extension contracture of the knee (full extension with an inability to flex past 30°) with a modifier for knee status: located (A) and dislocated (B). Type 3 is defined as a combined flexion and extension contracture (the rigid knee, flexion contracture >30°, <45° total arc of motion) with a modifier for quadriceps function: present (A) or absent (B) The absence of quad function was defined as 0/5 on clinical exam and presence of quad function was defined as 1–4/5 (from a quiver of motion to good strength over their restricted range) on clinical exam. No patients in our series had 5/5 quadriceps. A hip modifier was given to each patient based on the presence of: F-hip flexion/abduction contracture (>30°), E-hip extension contracture (<70° of flexion) or N-normal (Table 1).

Patients were included in the study if they were classified as type 1 or type 3 and underwent surgical treatment for their knee flexion deformity. Inclusion criteria for clinical follow up included a minimum period of 6 months, availability of pre- and post-operative range of motion (ROM) and ambulatory/assistance status. Exclusion criteria included patients with extension contractures (type 2) and patients who were diagnosed with pterygium or pterygium-like syndrome.

Patient demographics, surgical history, range of motion (ROM) and ambulatory status were recorded from the chart and physical therapy notes. The amount of shortening was recorded from the operative note. ROM measurements were recorded at three time points: preoperative visit, at the first clinical and physical therapy visit following surgery and at the last clinical follow-up. Patient’s ambulatory status, bracing, and use of assistive devices were recorded pre-operatively and at the time of last follow-up. The ambulatory status was recorded for each patient as either ambulatory or non-ambulatory at each timepoint. Ambulatory patients included both home or community ambulators while non-ambulatory patients included non-functional ambulators and non-ambulatory as defined by Hoffer’s classification [13]. Patients were considered independent community ambulators if able to walk in the community with or without aids. Household ambulators could walk in the home with aids but required a wheelchair in the community. Non-functional ambulators always required a wheelchair but could perform transfers and ambulated during physical therapy and the non-ambulators always required wheelchairs and could not perform transfers.

Statistical analysis was performed using GraphPad Prism 9.0.0 for Mac (Graphpad Software, San Diego, CA, USA). Knee range of motion and flexion deformity were analyzed using a Friedman test and multiple comparisons were performed using a Dunn’s post hoc analysis. A mixed effects model with Sidak’s post-hoc was used for the sub analysis on time of follow up and degree of flexion contracture. Descriptive analyses were used to compare ambulation pre-operatively and at last follow-up as non-ambulatory or ambulatory (with or without assistive devices).

## 3. Surgical Technique

**Figure 1:**
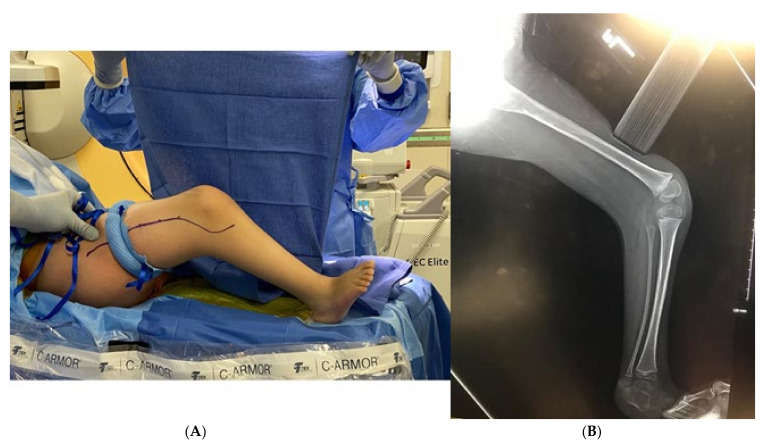
(**A**) Example of incision placement on the lateral thigh. (**B**) Maximum extension radiograph demonstrating 70° knee flexion contracture.

**Figure 2:**
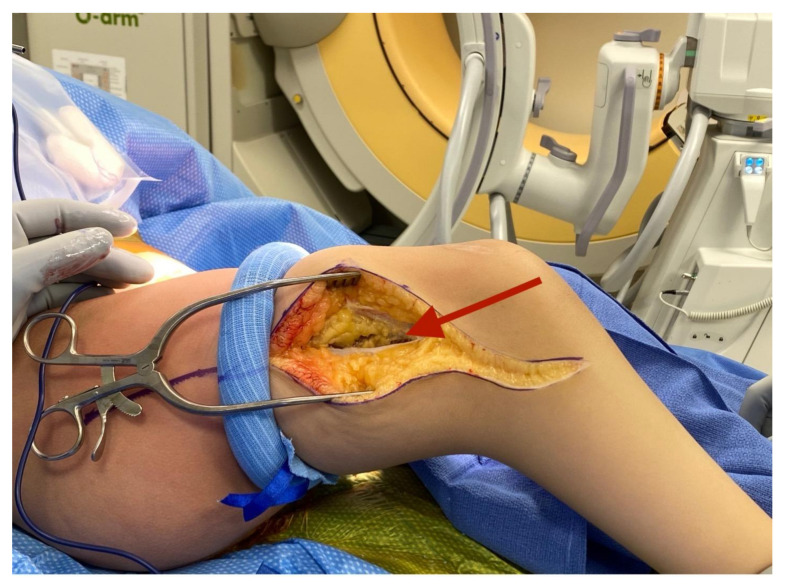
Iliotibial band incised and raised off quadriceps membrane.

**Figure 3:**
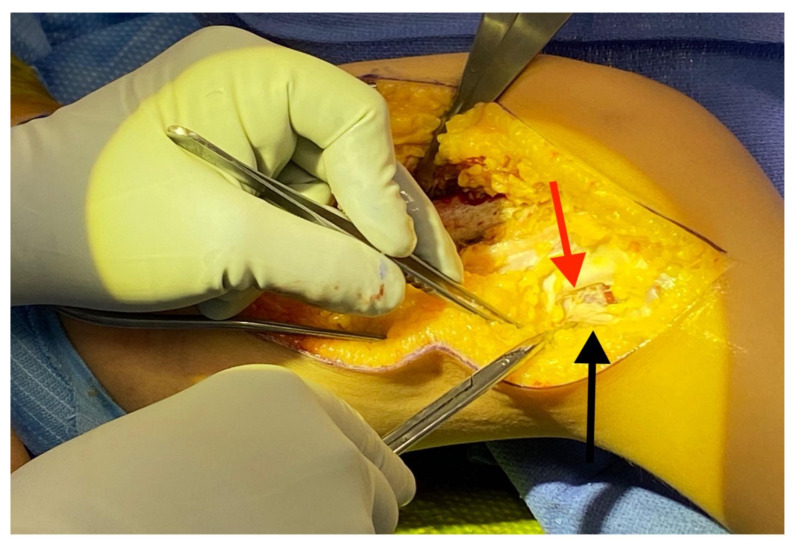
Isolation of the common peroneal nerve (red arrow) under the biceps femoris (black arrow).

**Figure 4:**
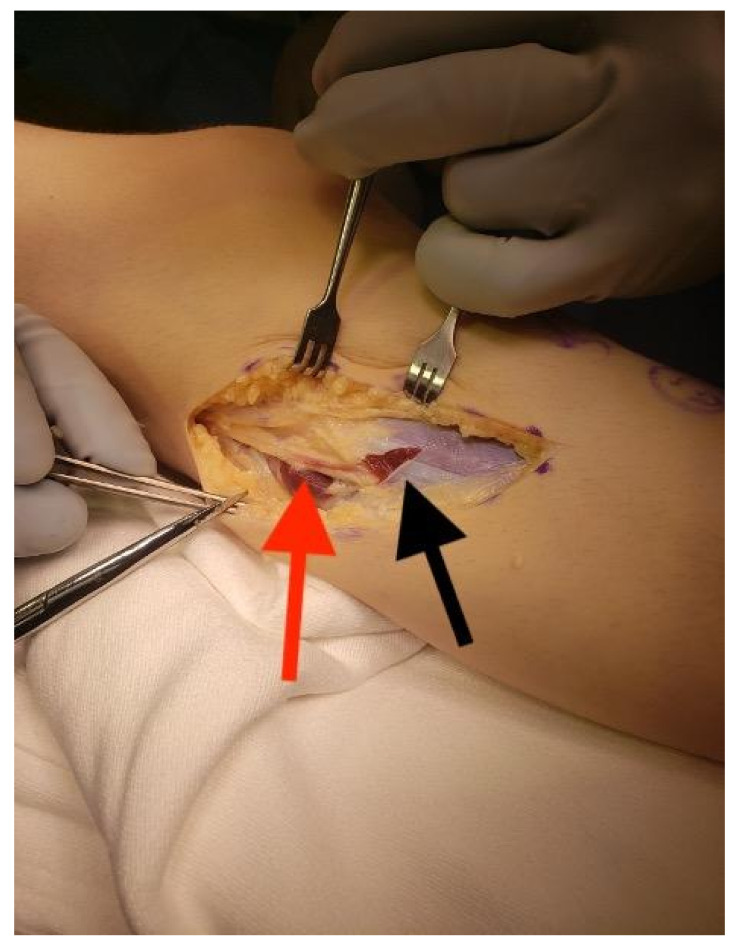
Peroneal nerve released (red arrow) as is crosses the fibula and enters the lateral compartment; note the start of the transverse fasciotomy of the lateral compartment that will continue through the anterior compartment (black arrow).

All Types 1A, 1B, 3A and 3 B patients received a standardized surgical treatment for knee flexion contractures which consisted of:

A tourniquet is applied. An incision is made from the anterior compartment of the leg directed postero-laterally across the knee with a line along the femur directed towards the subtrochanteric region of the femur up until the limit of the tourniquet (Figure 1A,B). The iliotibial band is incised and the posterior half (posterior to mid-axial line of the knee) of the iliotibial band is opened including the intermuscular septum to and including the periosteum (Figure 2). This release of the iliotibial band is often performed several times along the thigh. A subperiosteal Hohman retractor is placed around the midshaft of the femur and the femoral shaft is subperiosteally exposed raising the quadricep muscle (if present) anteriorly and distally to the level of the supracondylar region of the femur and proximally to the extent the tourniquet allowed. The common peroneal nerve is found as it exits the thigh under the biceps tendon (Figure 3). The biceps tendon is released. The peroneal nerve is decompressed proximally until it joins with the posterior neurovascular bundle. The nerve is freed and decompressed across the fibular neck and into the lateral compartment (Figure 4). A transverse fasciotomy is performed of the anterior and lateral compartments and the intermuscular septum is completely divided with care not to injure the transversing deep peroneal nerve and the nerve to the tibialis anterior. Any restriction of the peroneal and sciatic nerve will cause post-operative pain and dysfunction and is the reason for the decompression.

**Figure 5:**
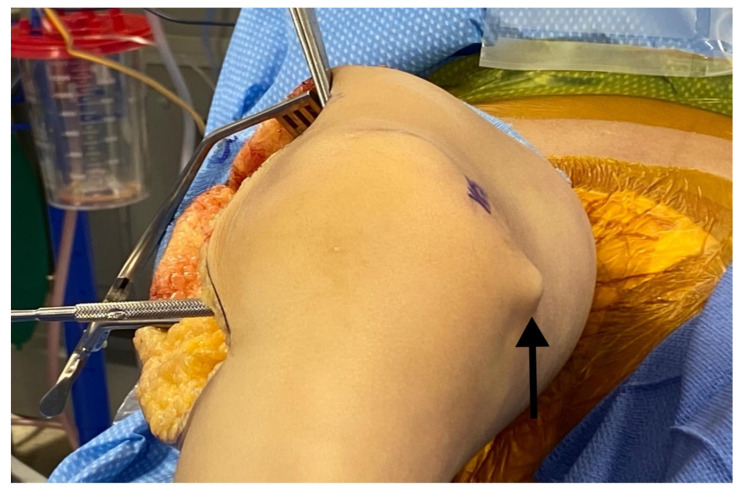
Freer elevator from lateral side demonstrating location of medial incision.

**Figure 6:**
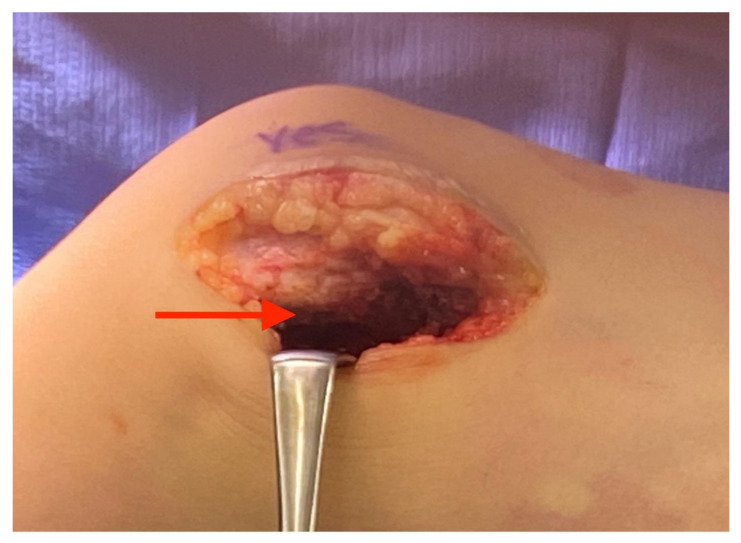
Medial incision with release of the postero-medial capsule of the knee.

Attention is turned to the posterior knee. The gastrocnemius is released off the postero-lateral femur with a cautery and sharp dissection and, if possible, blood vessels to the posterior femur are left intact. It is important to not drift distally passed the knee joint as the vascular bundle will be at risk. The capsule is noted and is sharply opened postero-laterally leaving the lateral collateral ligament intact. The capsule is opened posteriorly and the middle geniculate artery is noted and often spared. It may be coagulated with a bipolar cautery if needed for exposure. A freer elevator is then placed from lateral to medial across the back of the knee and a counter 5 cm medial incision is then made where the skin is tented by the elevator on the medial side (Figure 5). The saphenous nerve is found and protected. The fascia and hamstring tendons are released. The medial gastrocnemius is then released from the distal femur with a cautery and sharp dissection and again, if possible, the blood vessels to the distal femur are left intact. The medial capsule is then sharply opened and released posteriorly connecting to the posterolateral capsulotomy (Figure 6). The posterior cruciate ligament is left intact unless felt to be preventing full knee extension. The anterior cruciate ligament is often transected when present to prevent subluxation of the knee posteriorly as the knee is extended. A freer elevator is placed from posterior to anterior around the medial and lateral femoral condyles separately, indirectly releasing any anterior knee adhesions. At this point the knee should extend about 30–40° better than before the procedure but the peroneal nerve and vascular bundle may be quite tight with attempted extension. The tourniquet is removed, and the distal wound is covered with moist gauze and wrapped.

**Figure 7:**
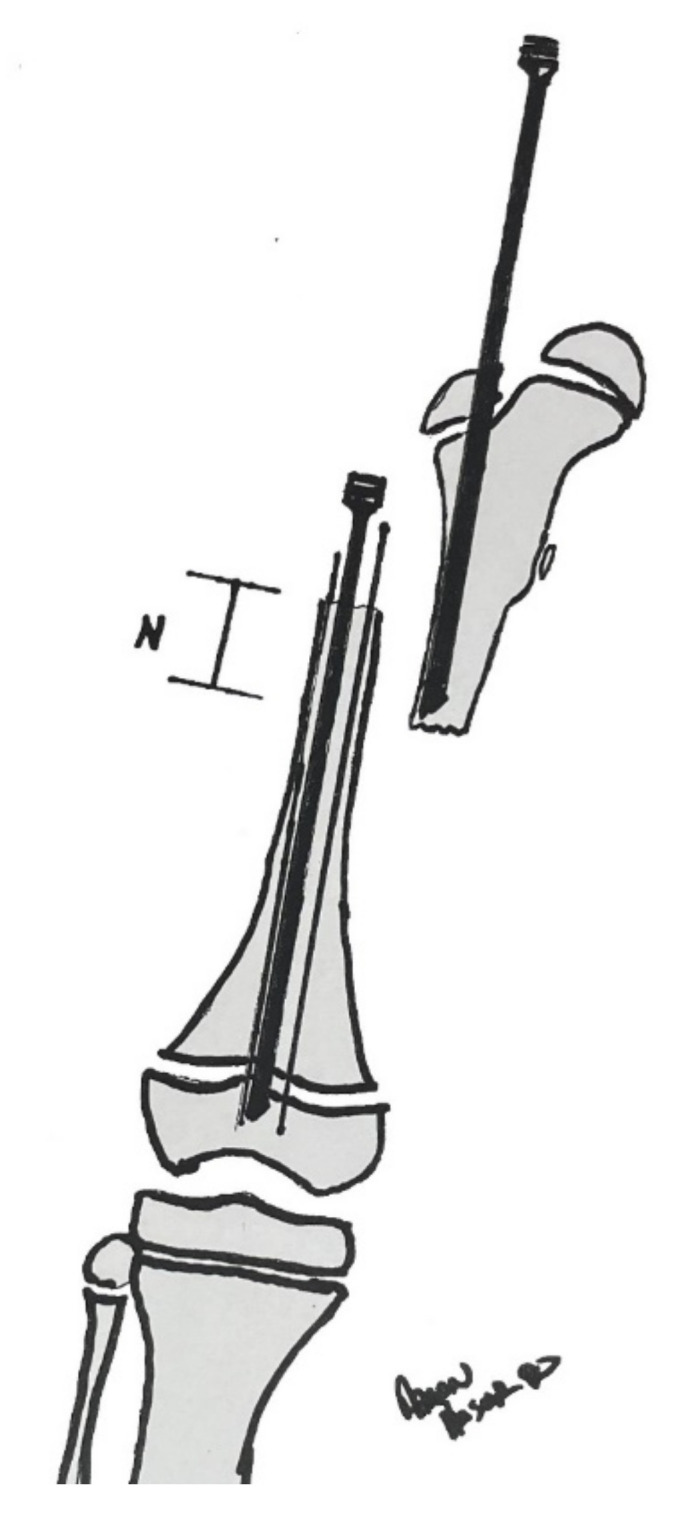
Drawing of the rod in the proximal femoral fragment and a rod in the distal femoral fragment crossing the distal physis. There are two 1.8 or 1.5mm wires also traversing the distal fragment and through the physis to prevent an iatrogenic fracture. Notice the distal fragment is allowed to migrate proximally to decrease tension on the neurovascular bundle; this distance is denoted as “N”. The knee will now be gently extended and the amount “N” will be the amount removed from the distal fragment for the shortening. Copyright Aaron Huser, DO.

**Figure 8:**
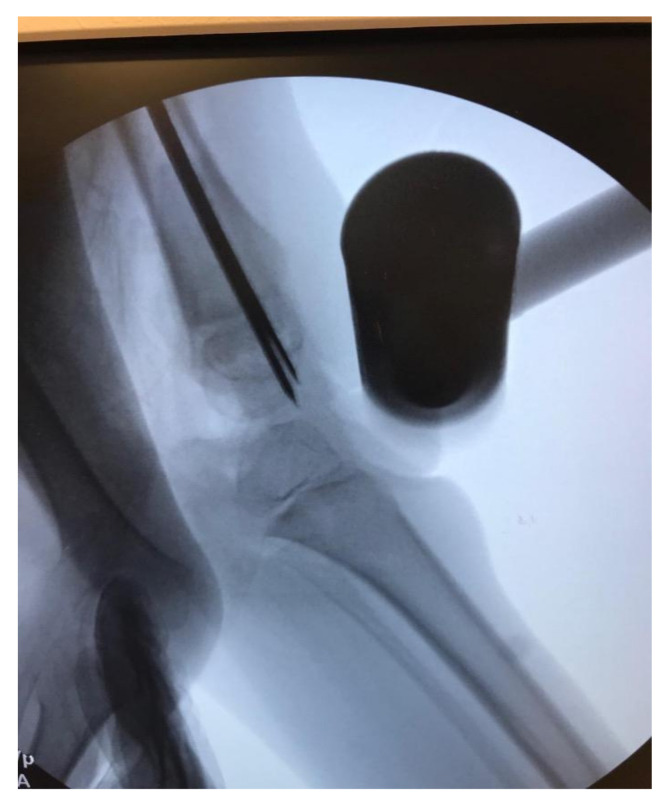
Fluoroscopic view of the knee in max extension after the proximal osteotomy; notice the wires and rod crossing the physis to protect it while the knee is extended.

**Figure 9:**
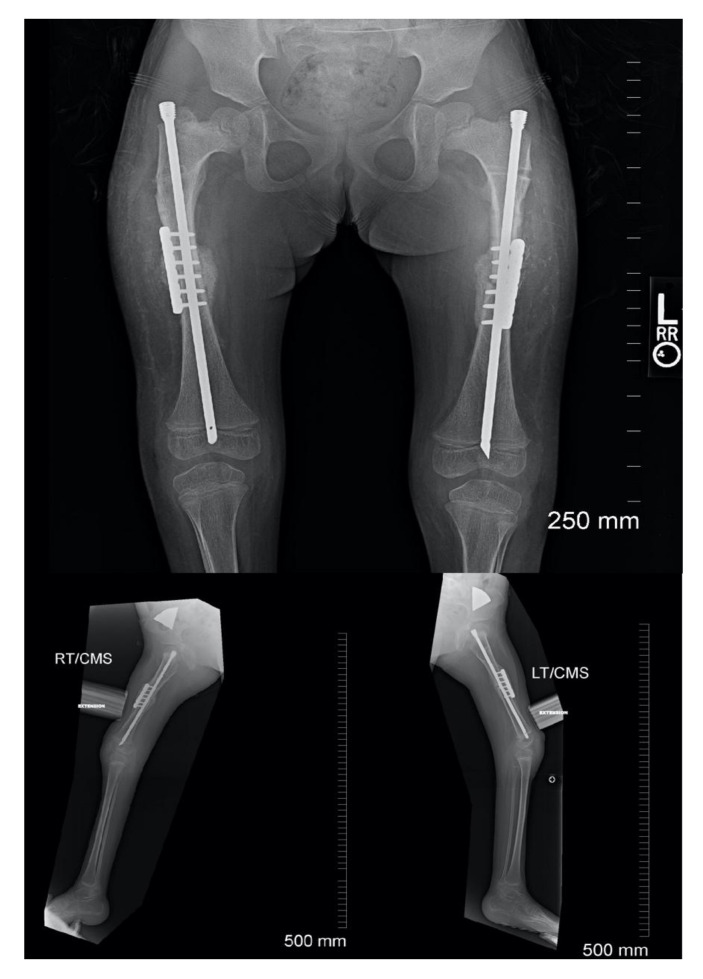
Post-operative follow-up demonstrating rod and plate construct as well as healing of osteotomy.

The incision is extended proximally to the subtrochanteric region and the remainder of the proximal femur is subperiosteally dissected. A wire for the appropriate, predetermined, diameter Slim™ rod (Pega Medical, QC, Canada) is inserted from the greater trochanter past the distal femoral physis. The canal is reamed to prepare for placement of the final rod. The length of the rod is determined by measuring the amount of wire in the canal (using another wire and ruler) and subtracting the presumed amount of shortening. For example, if the distance from the greater trochanter to just past the distal femoral physis measures 250 mm and you plan on shortening 50 mm then the rod length should be 200 mm. If the rod length is found to be incorrect, it can be exchanged after shortening osteoplasty. A rotational line is created on the femur with a sagittal saw and the first rod is placed just proximal to the osteotomy site. The osteotomy is performed, and the distal end is delivered out of the wound with a lion jaw. A second rod is placed into the distal femoral segment and into the distal epiphysis of the femur. A 1.8 mm wire is inserted down the canal parallel to the Slim ™ rod into the epiphysis. This is done to protect the physis (Figure 7). Prior to extending the knee the ends of the osteotomy are overlapped to allow shortening and loosening of the soft tissues, particularly the neurovascular bundle. (Figure 7) The knee is extended slowly indirectly releasing adhesions until full extension is obtained. Full extension of the knee is confirmed on a fluoroscopic cross-table lateral (Figure 8), the distal rod and wires are removed, and the overlap of the distal femoral segment to the proximal segment is marked on the distal femoral segment. The femur is shortened the marked amount and should allow for full extension of the knee. This is usually between 3–5 cm but there are times when more may be needed. The proximal rod is inserted into the distal shortened fragment and passed into the epiphysis distally and into the greater trochanter proximally. Rotation is controlled with either a six to eight hole 2.0–2.7 locking plate placed anteriorly or posteriorly to the intramedullary rod (Figure 9). It can be helpful to obtain a lateral fluoroscopic view of the femur to determine optimal plate position relative to the rod. Most often the screws are bicortical. Rotation is checked to be certain that what was once thought to be a rotational deformity may not be. The femoral bone removed from the shortening is ground and used as graft at the osteotomy site. The wound is closed over a 7 mm JP drain.

If patients had a previous proximal femoral varus osteotomy with a resulting neck shaft angle of less than 100° then a 130° blade plate is placed instead of the Slim™/locking plate combination to correct the neck shaft angle and fix the shortening osteoplasty. A Slim™ rod is inserted from distally to allow early range of motion.

**Figure 10:**
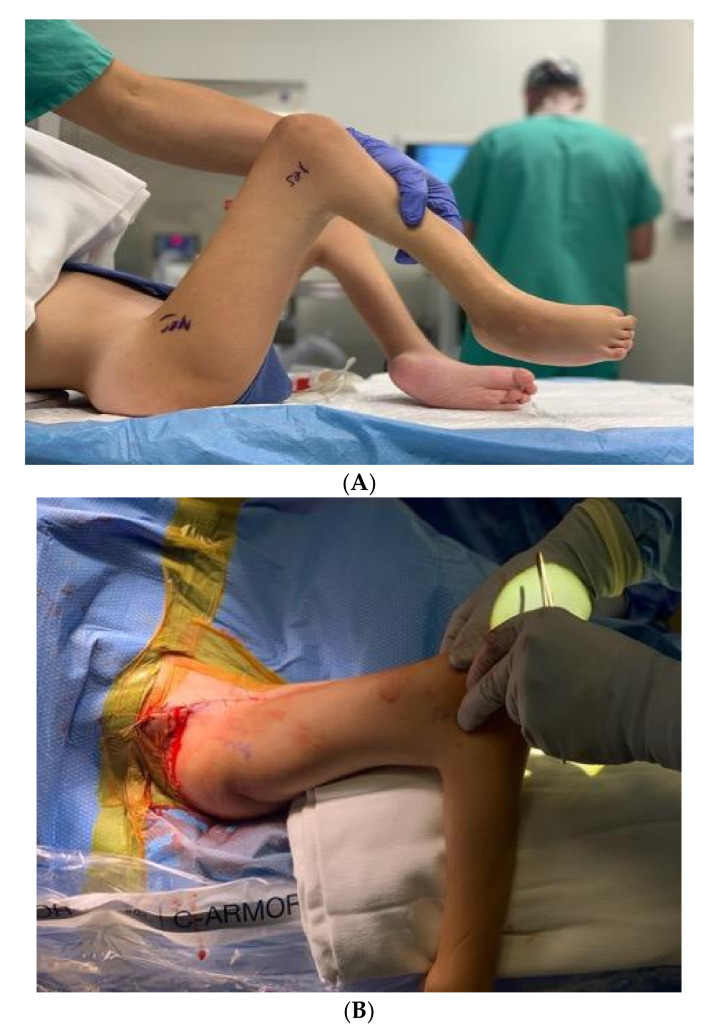
(**A**) Preoperative hip flexion contracture >70°; (**B**) post hip flexor release with less than 20° contracture.

In patients with a flexion/abduction contracture (F modifier), the hip is treated prior to the knee. An ilioinguinal incision is performed and the lateral femoral cutaneous nerve is protected in the interval between the sartorius and the tensor fascia lata. The apophysis is split with a 15 blade from the anterior inferior iliac spine directed posteriorly beyond the curve of the iliac wing. The abductor and flexor musculature are slid off the ileum. The iliac wing is shortened sufficiently to allow lengthening of the iliacus and abductors. If necessary, a proximal rectus recession was performed. If present, the iliopsoas undergoes a recession over the brim of the pelvis. (Figure 10A,B).

In patients with an extension contracture of the hip (E modifier). The femoral insertion of the gluteus maximus is released and the hamstrings are released proximally off the ischium. Neurolysis is performed of the sciatic nerve through the same incision.

**Figure 11:**
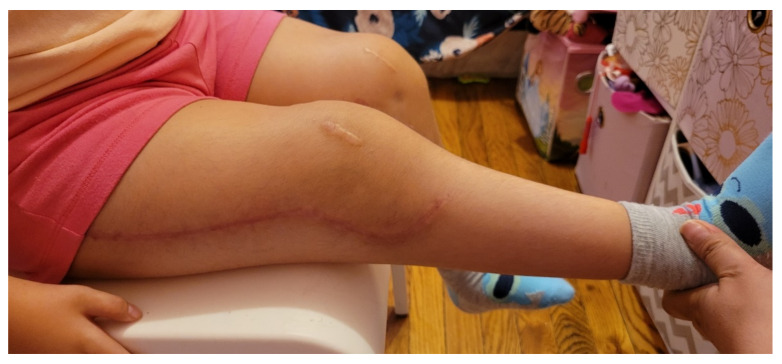
Clinical result of the patient from Figure 1.

Postoperatively, the patients are placed in a removable knee immobilizer. Physical therapy and range of motion begins approximately 3 to 7 days after surgery. Physical therapy consists of range of motion exercises with no limitations in order to achieve full extension as well as maintaining as much flexion as possible. At two weeks postoperatively, the patient is fitted for a KAFO with a ratchet hinge knee set anteriorly which they wear at night and for ambulation. If they have a F type hip, we also recommend prone lying as much as possible. Ambulation begins approximately 4 weeks postoperatively. The plate and screws are typically removed 6–12 months after the original operation. Figure 11 demonstrates the typical postoperative clinical result.

## 4. Results

We reviewed 70 patients with 135 knees. Of the 135 knees we excluded 44 knees that did not have surgery. Additionally, 18 knees were excluded because they were Type 2.

There were 39 patients (24M/15F) with 73 knees that remained for final evaluation (Table 2). The median age at the time of surgery was 5.2 years (range 1.7–13.9 years). The mean follow-up was 21 months (±12 months) with a minimum of 6 months. There were 64 type 1 and 9 type 3 knees included in the study. Intramedullary rod with plate rotational control was utilized in 54 femurs and proximal femoral blade plates were utilized in 19 legs.

The mean preoperative flexion contracture was 57° (±21°). The mean preoperative maximum flexion was 103° (±25°). The mean preoperative arc of motion was 45° (±22°). Post-operatively the mean flexion contracture was 4° ± 6° (*p <* 0.0001), mean maximum flexion was 74° ± 21° (*p* < 0.0001), and arc of motion was 71° ± 22° (*p* < 0.0001).

**Figure 12:**
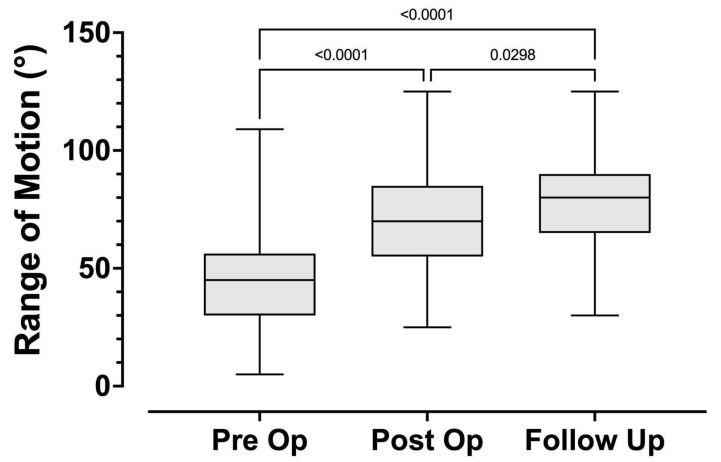
Knee range of motion comparison pre-operatively, post-operatively, and at follow-up for patients with a minimum of 6 month follow-up.

**Figure 13:**
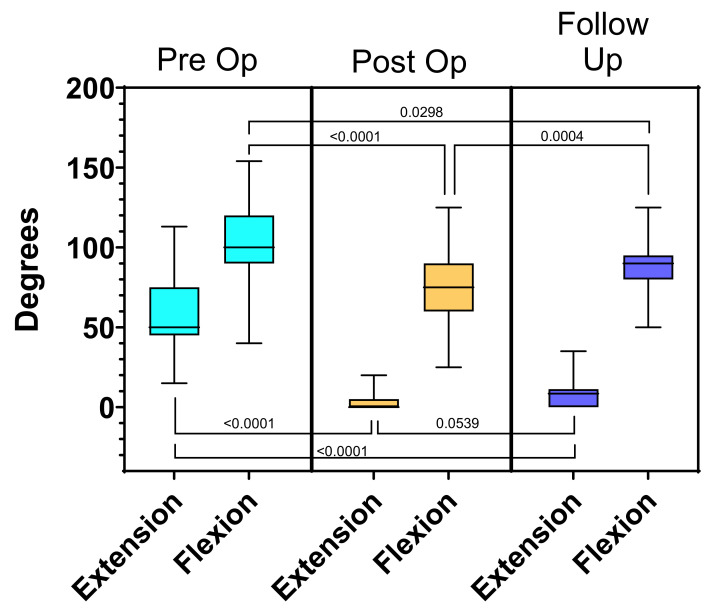
Demonstrates the flexion and extension values pre-operatively, post-operatively, and at follow up. The overall range of motion increased but the arc of motion also was shifted to a more functional range.

At final follow-up, the mean flexion contracture was 8^o^ (±9^o^) (*p* < 0.0001), mean maximum flexion was 87° (±15°) (*p* < 0.0001) and mean arc of motion was 79° (±19°) (*p* < 0.0001). Comparison of the preoperative and final follow-up measurements for flexion contracture, maximum flexion and arc of motion all demonstrate statistical significance and are summarized in Table 3 and Figure 12 and Figure 13. Data for the amount of femoral shortening was obtained for 61 limbs. The median amount of femoral shortening was 40mm (range 15–110 mm).

Sub-analysis was performed on the patients with 24 months or greater follow up. A two-way mixed effects model with Sidak’s post hoc test was used to compare pre-operative and follow-up range of motion between patients who had 24 months or greater follow up (n = 28 knees; mean follow up = 33 months) and those who had between 6 and 23 months of follow up (n = 45 knees; mean follow up = 14 months). There was no significant difference between the groups, indicating that range of motion was maintained (*p* = 0.0689). Last followup mean flexion contracture was 7° in the <24 month follow-up group and 9° in the 24 or more month follow-up group. (*p* = 0.4474) Average range of motion in the 6–23 month group was 46° (±24°) pre-operatively and 82° (±20°) at follow-up, and the 24 + month group was 43° (±17°) pre-operatively and 74° (±17°) at follow up.

**Figure 14:**
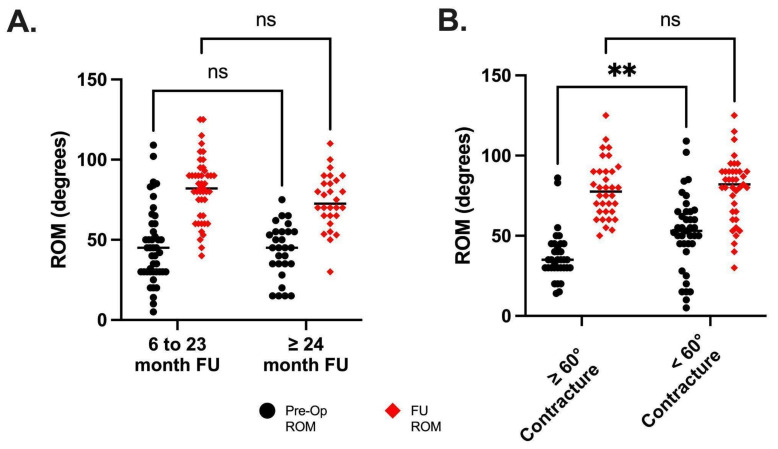
Sub analysis of ROM for patients with 6–23 months of follow up vs. ≥24 months of follow up (**A**). There were no differences in either pre- or post-operative range of motion, indicating that 6 months of follow up is sufficient to determine the effectiveness of treatment. (**B**) shows the sub analysis of range of motion for the patients who started with a flexion contracture of ≥60°. ** The range of motion was less before surgery but there was no difference in ROM post-surgery, indicating that even the patients with large flexion contractures reached a similar range of motion post-operatively.

Additional sub-analysis was performed on patients who presented with >60° flexion contracture pre-operatively. A two-way mixed effects model with Sidak’s post hoc test was used to compare total range of motion pre-operatively and at follow up for those with <60° flexion contracture (n = 39 knees; mean flexion contracture = 41°) and with 60° and greater flexion contracture (n = 34 knees; mean flexion contracture = 78°). Total range of motion was impacted significantly by the degree of preoperative contracture (*p* = 0.0268), meaning that patients with a higher degree of contracture had a less ROM pre-operatively. As well, the surgery (*p* < 0.0001) increased the range of motion and the greater the contracture the more the ROM increased (*p* = 0.0248). Post hoc analysis shows a difference in total range of motion pre-operatively (*p* = 0.0039) and no difference at follow up (*p* = 0.9997). Although the total range of motion improved in both groups it improved more in the group with >60° flexion contracture, thus explaining the interaction effect. In the >60° flexion contracture group the range of motion improved from an average of 37° ± 15° to 79° ± 18° and in the <60° flexion contracture group the range of motion improved from an average of 52° ± 24° to 79° ± 20°. These results are summarized in Table 4 and Figure 14.

Hip surgery was performed in 29 of 73 limbs. There were six hips in four patients with extension (E) type modifiers that underwent release of the gluteus maximus off the femoral shaft, sciatic nerve neurolysis and proximal hamstring release. The mean preoperative flexion contracture was 2° (0–5°) and mean flexion was 18° (0–45°). The mean arc of hip motion was 16° (0–45°). At last followup, the mean flexion contracture was 0° and mean flexion and arc of motion was 57° (35–65°). Twenty-four hips in fourteen patients with flexion (F) type hip modifiers underwent iliac wing shortening, relative abductor lengthening, iliopsoas lengthening and proximal rectus femoris release. Data was available for analysis of 19 of the hips. The mean preoperative hip flexion contracture was 50^o^ (25–70°). At last followup, the mean flexion contracture was 5° (0–25°) and mean arc of motion was 83° (60–100°).

**Figure 15:**
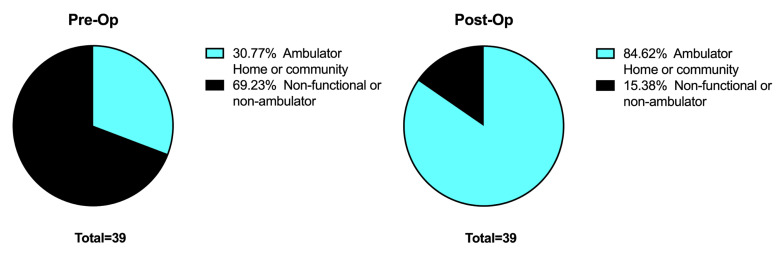
Ambulation pre and post treatment.

Pre-operative ambulation included 12 ambulators (31%) and 27 non-ambulators (69%). At follow-up there were 33 ambulators (84%) and 6 non-ambulators (16%) (Figure 15). Of the 33 ambulators, 21 were using knee-ankle-foot orthoses and 12 were using ankle-foot orthoses.

Complications include three non-unions of proximal femoral revisions, two diaphyseal femur fractures, one supracondylar/Salter–Harris I distal femur fracture, one temporary peroneal nerve palsy, and three wound breakdowns. The three proximal femoral non-unions all occurred in patients who were undergoing revision surgery. They were all treated with open repair of non-union and went on to heal after the secondary procedure. One of the diaphyseal femur fractures occurred when we were only fixing the osteotomy with a plate and screw construct. This complication led to the addition of an intramedullary rod to all of our constructs. The other diaphyseal femur fracture occurred with a rod in place and was treated non-operatively. The supracondylar/Salter–Harris I distal femur fracture occurred intraoperatively during extension of the knee. This was treated with pinning and splinting for three weeks, and then resumption of physical therapy. The complication led to the addition of multiple 1.5/1.8mm wires placed antegrade through the distal femoral fragment in the distal femoral epiphysis (as well as the rod) prior to performing the extension correction. These wires are removed prior to fixation of the femur. There have been no signs of growth arrest or bar formation in this patient’s femur. The temporary peroneal nerve palsy was treated non-operatively and motor and sensation function fully returned at 8 months. The three wound breakdowns were treated with debridement and delayed primary, closure. All complications resolved with the appropriate treatment. No patients complained of pain, instability or experienced growth arrest of the distal femur at latest followup.

## 5. Discussion

The ability to achieve an increased range of motion in patients with AMC and a functional range of motion has not been previously demonstrated in the literature [4,7,12]. Distal femoral extension osteotomies, which have had success in patients with cerebral palsy, have also been attempted in AMC [5,6,14,15,16]. In AMC, the supracondylar osteotomies improved ambulation ability, but did not improve total range of motion and had issues with recurrence of the deformity [5,6,14]. That being said, there may be a role for the distal femoral supracondylar osteotomy in patients who are skeletally mature, have knee flexion >90° and a mild amount of deformity (<20°). The use external fixation is also successful in achieving extension but has been fraught with causing a stiff knee [4,7,8,9,10,12]. Overall, improvement of ambulation ability has been demonstrated with knee surgery in AMC [14]; however, the ability to increase the total arc of motion has not be shown.

The goals of this study were to achieve full extension, increase the total range of motion and improve ambulatory function. This study reviewed a specific type of treatment in the management of AMC flexion contractures of the knee with or without hip flexion contracture. The results demonstrated a single, acute correction can obtain full extension, increase range of motion (34°), and maintain nearly full extension at follow-up. Flexion was maintained and most patients improved their level of ambulatory function.

The procedure is novel in combining posterior knee release, neurolysis and proximal femoral shortening with early postoperative range of motion exercises to achieve the desired results. Femoral shortening has been described in the literature by Saleh et al. [17] in treating popliteal webbing syndrome and mentioned by Ho et al. [9] in treating arthrogryposis, however, no mention is given where on the femur the shortening occurs, and the outcome in these patients. Shortening, in general, is required to achieve full knee extension. Prior to shortening, all structures have been released or lengthened except for the nerves, arteries and veins. It would be unwise to lengthen or cut these structures. Instead, the femur is shortened and the nerves, arteries and veins are relatively lengthened. Proximal femoral shortening allows for a better lever arm to extend the knee intraoperatively, does not disrupt the patella femoral mechanism and allows for rotational control with good fixation to allow for early ROM.

The degree of contracture did not impact our outcome. Even in severe deformities of greater than 60°, we were able to achieve our stated goals. Two year follow-up in this group showed maintenance of the ROM.

Hip abduction and or flexion contractures were treated simultaneously and did not require proximal femoral osteotomies for reorientation [18]. We postulated that once the knee was straightened the hip deformity would not recur as the legs were now able to lie flat in position. This was proven to be true in follow up.

The classification utilized was helpful both for the treatment to be rendered and the outcome in regard to ambulation and the need for the type of orthotics. For example, patients with no quadriceps function prior to release of the knee will require a Knee-ankle-foot orthosis (KAFO) for independent ambulation [3]. We did find several patients who we thought had no pre-surgical quadricep function were able to ambulate with only an AFO. These patients had ⅖ quadricep function and that was sufficient to support the knee in ambulation. We excluded type 2 (extension contractures) from this study due to small numbers and inconsistent treatment for these deformities making conclusions regarding management difficult.

We also noticed a varying degree of posterior subluxation under fluoroscopy following the acute extension in the operating room. We do not know the effect or cause of the radiographic posterior subluxation of the knee at maximum extension. This requires further investigation into its clinical significance. However, this may be part of the reason why a vast majority of our patients have 5–10° flexion contracture recurrence

This study is not without its limitations. First, this is retrospective study and our data is derived from what is available in the medical records. This was most prevalent when assessing improvements following hip surgery. Second, we do not have any patient reported outcomes or results from functional testing. Although we have shown that we were able to increase motion and more patients were able to walk after surgery, we do not know how patients/families feel about this operation and postoperative physical therapy requirements. Third, the data we have presented is for clinical outcomes after at least six months and in a smaller cohort, clinical outcomes after at least 24 months. Longer term follow-up is necessary as patients with arthrogryposis are known to experience recurrence of their contractures.

Moving forward, we plan on collecting data prospectively with the addition of patient reported outcomes to aid our understanding of the patient experience and functional outcomes. Additionally, we hope to answer questions on the ideal age for performing the procedure, develop an algorithm to better determine the amount of shortening required, assess for long-term sequelae such as pain and monitor to for recurrences.

## 6. Conclusions

Arthrogrypotic hip and knee flexion contractures can be treated safely in a single stage, improving range of motion and ambulation. The classification for the knee and hip allows the treating physician to outline a surgical plan for the hip and knee and prognosticate regarding ambulation and the need for bracing. This study demonstrates that it is possible to achieve a functional range of motion of the knee and hip in patients with AMC while improving ambulatory function. Because flexion of the knee remains largely unimpacted, seating is not altered and is likely improved. While no one procedure can be perfect in achieving function in this difficult patient population, the results demonstrate that good function is met without further significant intervention.

## Figures and Tables

**Table 1:** Knee classification according to type of contracture. Each knee type has an A or B modifier as well as a hip modifier.

Arthrogryposis Knee Classification					
Type 1		Type 2		Type 3	
Flexion Contracture		Extension Contracture		Flexion and Extension Contracture	
Type 1 modifiers		Type 2 modifiers		Type 3 modifiers	
1A	1B	2A	2B	3A	3B
Quadriceps present	Quadriceps absent	Knee located	Knee dislocated	Quadriceps present	Quadriceps absent
Hip Modifiers					
F = hip flexion or abduction contracture (>30°)					
E = hip extension contracture (flexion <70°)					
N = Normal					

**Table 2:** Distribution of patients analyzed by knee classification.

Arthrogryposis Knee Classification			
Type 1		Type 3	
Flexion Contracture		Flexion and Extension Contracture	
1A	1B	3A	3B
25	39	2	7
Hip Modifiers			
F: 2	F: 18	F: 0	F: 4
E: 3	E: 3	E: 0	E: 0
N: 20	N: 18	N: 2	N: 3

**Table 3:** Range of Motion Values.

	Time Point			Multiplicity Adjusted *p*-Value		
	Pre-Op	Post-Op	Follow-Up	Pre- vs. Post-	Post vs. Follow-Up	Pre- vs. Follow-Up
Flexion Contracture	57° ± 21°	4° ± 6°	8° ± 9°	<0.0001	0.0539	<0.0001
Maximum Flexion	103° ± 25°	74° ± 21°	87° ± 15°	<0.0001	0.0004	0.0298
Range of motion	45° ± 22°	71° ± 22°	79° ± 19°	<0.0001	0.0298	<0.0001

**Table 4:** ROM values for sub analysis of length of follow up and flexion contractures.

	Range of Motion		*p*-Value
Time Period	6–23 mo. (*n* = 45)	≥ 24 mo. (*n* = 28)	
Pre-op	46° ± 24°	43° ± 17°	0.6758
Follow-up	82° ± 20°	74° ± 17°	0.1400
Flexion Contracture	<60° (*n* = 39)	≥60° (*n* = 34)	
Pre-op	52° ± 24°	37° ± 15°	0.0039
Follow-up	79° ± 20°	79° ± 18°	0.9997

## Data Availability

Data for this study can be found by contacting the corresponding author via email: dfeldman@paleyinstitute.org.

## References

[1] Hall J.G. (2014). Arthrogryposis (multiple congenital contractures): Diagnostic approach to etiology, classification, genetics, and general principles. Eur. J. Med. Genet..

[2] Lowry R.B., Sibbald B., Bedard T., Hall J.G. (2010). Prevalence of multiple congenital contractures including arthrogryposis multiplex congenita in Alberta, Canada, and a strategy for classification and coding. Birth Defects Res. Part A Clin. Mol. Teratol..

[3] Donohoe M., Pruszcynski B., Rogers K., Bowen J.R. (2019). Predicting Ambulatory Function Based on Infantile Lower Extremity Posture Types in Amyoplasia Arthrogryposis. J. Pediatr. Orthop..

[4] Herzenberg J.E., Davis J.R., Paley D., Bhave A. (1994). Mechanical distraction for treatment of severe knee flexion contractures. Clin. Orthop. Relat. Res..

[5] DelBello D.A., Watts H.G. (1996). Distal femoral extension osteotomy for knee flexion contracture in patients with arthrogryposis. J. Pediatr. Orthop..

[6] Moreira M.V., Rimoldi A.C., Aoki S. (2014). Analysis on the results from percutaneous extensor osteotomy of the distal femur in patients with amyoplasia. Rev. Bras. Ortop..

[7] Damsin J.-P., Ghanem I. (1996). Treatment of Severe Flexion Deformity of the Knee in Children and Adolescents Using the Ilizarov Technique. J. Bone Jt. Surg. Br. Vol..

[8] Heydarian K., Akbarnia B.A., Jabalameli M., Tabador K. (1984). Posterior Capsulotomy for the Treatment of Severe Flexion Contractures of the Knee. J. Pediatr. Orthop..

[9] Ho C.A., Karol L.A. (2008). The Utility of Knee Releases in Arthrogryposis. J. Pediatr. Orthop..

[10] Lampasi M., Antonioli D., Donzelli O. (2012). Management of knee deformities in children with arthrogryposis. Musculoskelet. Surg..

[11] Palocaren T., Thabet A.M., Rogers K., Holmes L., Donohoe M., King M.M., Kumar S.J. (2010). Anterior Distal Femoral Stapling for Correcting Knee Flexion Contracture in Children with Arthrogryposis—Preliminary Results. J. Pediatr. Orthop..

[12] Van Bosse H.J.P., Feldman D.S., Anavian J., Sala D.A. (2007). Treatment of Knee Flexion Contractures in Patients with Arthrogryposis. J. Pediatr. Orthop..

[13] Hoffer M., Feiwell E., Perry R., Perry J., Bonnett C. (1973). Functional Ambulation in Patients with Myelomeningocele. J. Bone Jt. Surg..

[14] Yang S.S., Dahan-Oliel N., Montpetit K., Hamdy R.C. (2010). Ambulation Gains After Knee Surgery in Children with Arthrogryposis. J. Pediatr. Orthop..

[15] Novacheck T.F., Stout J.L., Gage J.R., Schwartz M.H. (2009). Distal Femoral Extension Osteotomy and Patellar Tendon Advancement to Treat Persistent Crouch Gait in Cerebral Palsy: Surgical Technique. J. Bone Jt. Surg..

[16] Rutz E., Gaston M.S., Camathias C., Brunner R. (2012). Distal Femoral Osteotomy Using the LCP Pediatric Condylar 90-Degree Plate in Patients with Neuromuscular Disorders. J. Pediatr. Orthop..

[17] Saleh M., Gibson M.F., Sharrard W.J.W. (1989). Femoral Shortening in Correction of Congenital Knee Flexion Deformity with Popliteal Webbing. J. Pediatr. Orthop..

[18] Van Bosse H.J.P., Saldana R.E. (2017). Reorientational Proximal Femoral Osteotomies for Arthrogrypotic Hip Contractures. J. Bone Jt. Surg..

